# Bifidobacterium animalis Promotes the Growth of Weaning Piglets by Improving Intestinal Development, Enhancing Antioxidant Capacity, and Modulating Gut Microbiota

**DOI:** 10.1128/aem.01296-22

**Published:** 2022-10-27

**Authors:** Jiaman Pang, Yisi Liu, Luyuan Kang, Hao Ye, Jianjun Zang, Junjun Wang, Dandan Han

**Affiliations:** a State Key Laboratory of Animal Nutrition, College of Animal Science and Technology, China Agricultural University, Beijing, China; b Adaptation Physiology Group, Department of Animal Sciences, Wageningen University and Research, Wageningen, The Netherlands; University of Helsinki

**Keywords:** *Bifidobacterium animalis*, gut microbiota, intestinal development, weaning piglets

## Abstract

Probiotics are widely used to promote performance and improve gut health in weaning piglets. Therefore, the objective of this study was to investigate the effects of dietary supplementation with Bifidobacterium animalis subsp. lactis (B. animalis) JYBR-190 on the growth performance, intestine health, and gut microbiota of weaning piglets. The results showed that the dietary addition of B. animalis significantly improved growth performance and decreased diarrhea incidence. B. animalis increased villus height in the duodenum and elevated goblet cell numbers and amylase activity in the jejunum. Additionally, B. animalis supplementation markedly increased total antioxidant capacity in jejunal mucosa but declined the malondialdehyde content. B. animalis treatment did not affect the mRNA expressions associated with the intestinal barrier and inflammatory cytokine in various intestinal segments. Microbiota analysis indicated that a diet supplemented with B. animalis significantly increased the relative abundances of health-promoting bacteria in the lumen, such as Streptococcus*, Erysipelotrichaceae*, *Coprococcus,* and *Oscillibacter.* There was a trend for B. animalis fed piglets to have a higher relative abundance of B. animalis in ileal digesta. Moreover, B. animalis-treated pigs decreased the abundance of *Helicobacter* and Escherichia-*Shigella* in ileal mucosa-associated microbiota. In summary, this study showed that B. animalis supplementation stimulated growth performance, improved gut development, enriched beneficial bacteria abundances, and declined intestinal pathogens populations, while B. animalis had limited effects on the intestinal barrier and immune function.

**IMPORTANCE** In the modern swine industry, weaning is a critical period in the pig’s life cycle. Sudden dietary, social, and environmental changes can easily lead to gut microbiota dysbiosis, diarrhea, and a decrease in growth performance. To stabilize intestinal microbiota and promote animal growth, antibiotics were widely applied in swine diets during the past few decades. However, the side effects of antibiotics posed a great threat to public health and food safety. Therefore, it is urgent to find and develop antibiotic alternatives. The growing evidence suggested that probiotics can be preferable alternatives to antibiotics because they can modulate microbiota composition and resist pathogens colonization. In this study, our results indicated that dietary supplementation with Bifidobacterium animalis promoted growth in weaning piglets by improving gut development, increasing beneficial bacteria abundances, and declining pathogens populations.

## INTRODUCTION

In the modern swine industry, weaning is a critical period in the pig’s life cycle. Piglets encounter multiple stressors during the postweaning period which cause disruptions of gut microbiota, intestinal inflammation, and diarrhea, consequently reducing their growth performance (1, 2). To stabilize intestinal microbiota and promote animal growth, antibiotics were widely applied in the swine diet in the past few decades (3, 4). However, the use of antibiotic growth promoters has been limited in pig production (3, 5). To maintain pig health during the postweaning period, it is urgent to find and develop antibiotic alternatives.

The mammalian gastrointestinal tract is inhabited by trillions of bacteria (6). A stable and well-balanced community of gut microbiota plays an important role in host health (2). Probiotics are living microorganisms when administered in adequate amounts, conferring a health benefit to the host (7). Supplementing probiotics in pig production can effectively modulate microbiota composition and inhibit pathogenic bacteria growth (8). In addition, probiotics can activate the immune system, resist pathogen colonization, and regulate intestinal barrier function (8, 9). Therefore, probiotics are considered desirable alternatives to benefit the weaning piglets (5, 10).

At present, the most frequently used commercial probiotics are *Lactobacillus*, *Bifidobacterium*, Streptococcus, and yeasts (10). A previous study has reported that *Bifidobacterium* enhanced gut health and immunity in weaning piglets (11). Meanwhile, the *Bifidobacterium* reduced the pathogen loads post-Salmonella challenge (12). Similarly, a recent report found that oral administration of Bifidobacterium animalis subsp. lactis (B. animalis) in germfree piglets can prevent Salmonella infection (13). Besides, B. animalis regulate inflammatory cytokines (14, 15), improve intestinal barrier function, and modulates gut microbiota (15). Moreover, *Bifidobacterium* species can improve their antioxidant capability (16, 17). However, the effects of B. animalis on the growth performance of weaning piglets have not been fully investigated. Therefore, we investigate whether B. animalis improve the growth performance of weaning piglets and then explore its underlying mechanisms.

## RESULTS

### Effects of Bifidobacterium animalis on growth performance of weaning piglets.

As shown in Table 1 to 3, dietary supplementation with B. animalis did not affect body weight (BW) on day 14 but significantly increased BW on day 28 (*P* < 0.05). No difference was found in average daily feed intake (ADFI) between the control group (Con) and the B. animalis group (Bif). Average daily gain (ADG) was increased in the group receiving the B. animalis-supplemented diet during days 15 to 28 and days 1 to 28 (*P* < 0.05). However, the feed conversion ratio (FCR) of piglets fed the B. animalis diet was decreased from day 15 to 28 and day 1 to 28 compared with piglets fed the control diet. Meanwhile, pigs fed the B. animalis diet markedly decreased the incidence of diarrhea (*P* < 0.05; Table 3).

**TABLE 1:** Composition of the experimental diets (as-fed basis)

Items	Phase 1, 1–14 day		Phase2, 15–28 day	
	Con	Bif	Con	Bif
Ingredient (%)				
Corn	62.10	61.85	67.27	67.07
Soybean meal 43%	10.00	10.00	15.00	15.00
Dehulled soybean meal 46%	5.00	5.10	2.00	2.05
Fish meal	5.00	5.00	3.00	3.00
Expanded soybean	6.00	6.00	6.00	6.00
Whey power	8.00	8.00	3.00	3.00
Soybean oil	0.35	0.40	0.35	0.40
CaHPO_4_	1.15	1.15	1.10	1.10
Limestone	0.70	0.70	0.60	0.60
Salt	0.20	0.20	0.20	0.20
Primix^*a*^	0.50	0.50	0.50	0.50
*L*-Lys-HCl (78.8%)	0.61	0.61	0.60	0.60
*DL*-Met (98%)	0.12	0.12	0.12	0.12
*L*-Thr (98%)	0.22	0.22	0.21	0.21
*L*-Trp (98%)	0.05	0.05	0.05	0.05
Bifidobacterium animalis^*b*^	0.00	0.10	0.00	0.10
Total	100.00	100.00	100.00	100.00
Calculated nutrient level (%)				
Digestible energy (MJ/kg)	14.44	14.44	14.48	14.48
Crude protein (%)	18.99	19.02	18.35	18.36
Total Ca (%)	0.88	0.88	0.72	0.72
Total P (%)	0.71	0.71	0.64	0.64
Lys (%)	1.35	1.35	1.28	1.28
Met (%)	0.40	0.40	0.38	0.38
Thr (%)	0.79	0.80	0.75	0.75
Trp (%)	0.23	0.23	0.23	0.23

a The premix provided the following per kg of diets: VA 12,000 IU, VD_3_ 2,500 IU, VE 30 IU, VK_3_ 3 mg, VB_5_ 10 mg, VB_12_ 27.6 μg, niacin 30 mg, choline chloride 400 mg, Mn (as MnO) 40 mg, Fe (as FeSO_4_·H_2_O) 90 mg, Zn (as ZnO) 100 mg, Cu (as CuSO_4_·5H_2_O) 8.8 mg, I (as KI) 0.35 mg, Se (Na_2_SeO_3_) 0.3 mg.

b Bifidobacterium animalis 10^10^ CFU per kg in the diet.

**TABLE 2:** The primers used in this study

Primer^*a*^	Forward 5′ → 3′	Reverse 5′ → 3′
Total bacteria	ACTCCTACGGGAGGCAGCAG	ATTACCGCGGCTGCTGG
*Bifidobacterium* spp	CGCGTCCGGTGTGAAAG	CTTCCCGATATCTACACATTCCA
*Lactobacillus* spp	GAGGCAGCAGTAGGGAATCTTC	GGCCAGTTACTACCTCTATCCTTCTTC
E. coli	CATGCCGCGTGTATGAAGAA	CGGGTAACGTCAATGAGCAAA
Salmonella spp	CCTTTCTCCATCGTCCTGAA	TGGTGTTATCTGCCCGACCA
*GAPDH*	ACCCAGAAGACTGTGGATGG	AAGCAGGGATGATGTTCTGG
*Mucin-1*	GTGCCGACGAAAGAACTG	TGCCAGGTTCGAGTAAGAG
*Mucin-2*	CTGTGTGGGGCCTGACAA	AGTGCTTGCAGTCGAACTCA
*ZO-1*	GCCATCCACTCCTGCCTAT	CGGGACCTGCTCATAACTTC
*Claudin-1*	AAGGACAAAACCGTGTGGGA	CTCTCCCCACATTCGAGATGATT
*Occludin*	CAGCAGCAGTGGTAACTTGG	CAGCAGCAGTGGTAACTTGG
*TNF-α*	CCACGCTCTTCTGCCTACTGC	TCGGCTTTGACATTGGCTACAA
*IL-1β*	CCGCCAAGATATAACTGAC	GCAGCAACCATGTACCAA
*IL-6*	AATGCTCTTCACCTCTCC	CACACTTCTCATACTTCTCAC
*IL-8*	TGTCAATGGAAAAGAGGTCTGC	CTGCTGTTGTTGTTGCTTCTCA
*IL-10*	GTCCGACTCAACGAAGAAGG	GCCAGGAAGATCAGGCAATA

a *GAPDH*, glyceraldehyde 3-phosphate dehydrogenase; *ZO-1*, zonula occludens-1; *TNF-α*, tumor necrosis factor α; IL, interleukin.

**TABLE 3:** The effects of Bifidobacterium animalis on the growth performance of weaning piglets

Items	Con^*a*^	Bif^*a*^	SEM^*a*^	*P* value
BW^*a*^, kg				
Day 1	8.16	8.16	0.03	0.944
Day 14	12.50	12.66	0.09	0.400
Day 28	19.05	19.66	0.14	0.022
ADG^*a*^, g				
Day 1–14	309.38	321.28	5.22	0.274
Day 15–28	468.15	500.02	7.28	0.020
Day 1–28	388.76	410.65	4.87	0.015
ADFI^*a*^, g				
Day 1–14	449.97	443.93	6.65	0.671
Day 15–28	777.78	785.91	7.92	0.631
Day 1–28	613.88	614.92	6.76	0.943
FCR^*a*^				
Day 1–14	1.45	1.38	0.02	0.066
Day 15–28	1.66	1.57	0.02	0.034
Day 1–28	1.58	1.50	0.02	0.008
Diarrhea incidence, %	12.20	6.85		0.018

a Con, Control group; Bif, Bifidobacterium animalis group; BW, body weight; ADG, average daily gain; ADFI, average daily feed intake; FCR, feed conversion ratio; SEM, standard error of the means. *n* = 6 for each group.

### Effects of Bifidobacterium animalis on immunological function and inflammatory cytokines of weaning piglets.

The concentrations of immunoglobulins (Ig) in serum, including IgA, IgG, and IgM, had no differences between the Con and Bif groups (Table 4). Similarly, there were no significant differences in serum of interleukin (IL)-1β, IL-6, and IL-10 between the two groups (Table 4).

**TABLE 4:** The effects of Bifidobacterium animalis on immunological function and inflammatory cytokines of weaning piglets

Items^*a*^	Con^*a*^	Bif^*a*^	SEM^*a*^	*P* value
IgA, mg/mL	0.85	0.91	0.05	0.569
IgG, mg/mL	6.91	6.28	0.21	0.141
IgM, mg/mL	0.64	0.77	0.05	0.147
IL-1β, pg/mL	28.27	29.14	1.21	0.735
IL-6, pg/mL	87.40	83.61	3.18	0.573
IL-10, pg/mL	32.03	30.55	2.97	0.811

a Con, Control group; Bif, Bifidobacterium animalis group; IgM, immunoglobulin M; IgG, immunoglobulin G; IgA, immunoglobulin A; IL-1β, interleukin-1β; IL-6, interleukin-6; IL-10, interleukin-10; SEM, standard error of the means. *n* = 7 for each group.

### Effects of Bifidobacterium animalis on antioxidant indicators of weaning piglets.

The antioxidant indicators as presented in Table 5. The antioxidant indicators in serum, including catalase (CAT), total superoxide dismutase (T-SOD), total antioxidant capacity (T-AOC), glutathione peroxidase (GSH-Px), and malondialdehyde (MDA) were not changed between the two groups. The addition of B. animalis to the diet did not influence the activities of CAT, T-SOD, and GSH-Px in the jejunum. However, B. animalis supplementation increased the activity of T-AOC (*P* < 0.05) and decreased the content of MDA in the jejunum (*P* < 0.05).

**TABLE 5:** The effects of Bifidobacterium animalis on antioxidant indices of weaning piglets

Items^*a*^	Con^*a*^	Bif^*a*^	SEM^*a*^	*P* value
Serum				
CAT, U/mL	2.58	3.08	0.23	0.301
T-SOD, U/mL	146.98	148.80	1.28	0.500
T-AOC, mmol/L	0.31	0.27	0.01	0.085
GSH-Px, U/mL	317.68	306.41	9.76	0.584
MDA, nmol/mL	4.23	3.96	0.13	0.335
Jejunum mucosa				
CAT, U/mg	1.17	1.16	0.10	0.971
T-SOD, U/mg	76.81	78.29	3.12	0.827
T-AOC, mmol/g	0.50	0.98	0.10	0.003
GSH-Px, U/mg	218.84	172.89	14.42	0.114
MDA, nmol/mg	1.63	0.92	0.18	0.037

a Con, Control group; Bif, Bifidobacterium animalis group; T-SOD, superoxide dismutase; CAT, catalase; GSH-Px, glutathione peroxidase; T-AOC, total antioxidant capacity; MDA, malondialdehyde; SEM, standard error of the means. *n* = 7 for each group in serum, *n* = 5 for each group in the jejunum.

### Effects of Bifidobacterium animalis intestinal morphology and barrier of weaning piglets.

**FIG 1:**
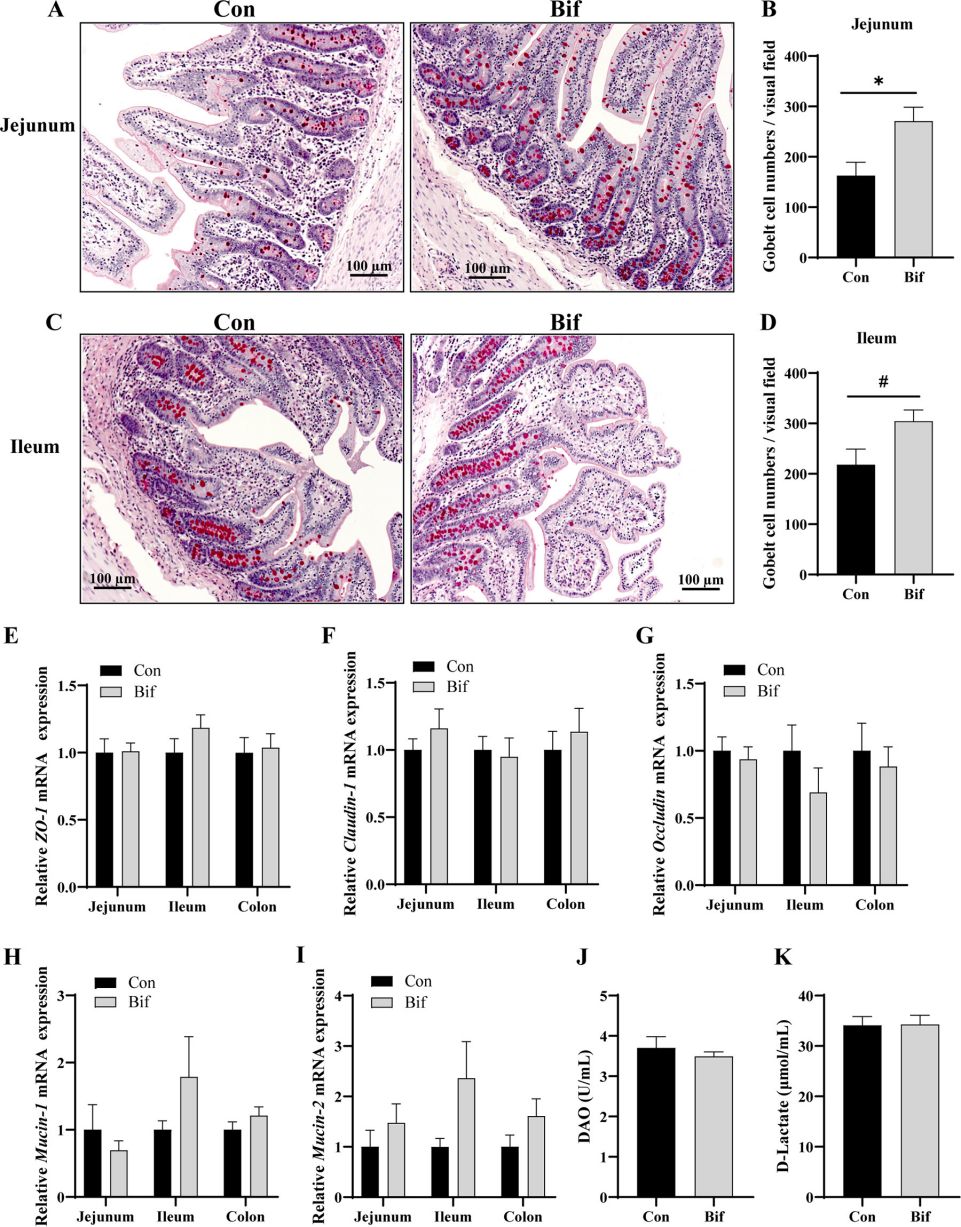
The effects of Bifidobacterium animali on the intestinal barrier. Goblet cells were stained red color by Periodic acid-Schiff (PAS) in the jejunum (A) and ileum (C) of weaning piglets; The number of goblet cells of the visual field in the jejunum (B) and ileum (D); (E to I) the relative mRNA expression of *ZO-1* (E), *Claudin-1* (F), *Occludin* (G), *Mucin-1* (H), and *Mucin-2* (I) in intestinal mucosa; the concentrations of DAO (J) and d-Lactate (K) in serum. DAO, diamine oxidase; *ZO-1*, zonula occludens-1. SEM, standard error of the means. *, *P < *0.05; #, significant trend. *n* = 7 for each group in serum samples; *n* = 5 for each group in the intestinal tissue samples.

The intestinal morphology and barrier are shown in Table 6 and Fig. 1. There were no effects on villus height (VH), crypt depth (CD), and VH:CD ratio between the Con and Bif groups in the jejunum and ileum. The B. animalis treatment improved the VH and VH:CD ratio in the duodenum compared with the Con group (*P* < 0.05). In addition, supplementation of B. animalis to the weaning piglet's diet significantly increased the jejunal goblet cell numbers (*P* < 0.05) (Fig. 1A and B). The B. animalis supplementation tended to have higher goblet cell numbers in the ileum (*P = *0.054, Fig. 1C and D).

**TABLE 6:** The effects of Bifidobacterium animalis on intestinal morphology of weaning piglets

Items^*a*^	Con^*a*^	Bif^*a*^	SEM^*a*^	*P* value
Duodenum				
VH, μm	317	407	16.51	<0.001
CD, μm	284	281	6.44	0.842
VH:CD	1.12	1.46	0.06	<0.001
Jejunum				
VH, μm	414	418	6.15	0.732
CD, μm	282	297	12.92	0.592
VH:CD	1.47	1.45	0.07	0.873
Ileum				
VH, μm	289	312	6.90	0.085
CD, μm	198	189	6.66	0.504
VH:CD	1.47	1.66	0.06	0.091

a Con, Control group; Bif, Bifidobacterium animalis group; VH, villus height; CD, crypt depth; SEM, standard error of the means. *n* = 5 for each group.

No differences were observed for relative mRNA expression of tight junction molecules (including *zonula occludens-1* [*ZO-1*], *claudin-1*, and *occludin*) in the jejunum, ileum, and colon between the two groups (Fig. 1E to G). Similarly, there were no differences between the B. animalis-supplemented and nonsupplemented groups for the intestinal mucus barrier proteins (*mucin-1* and *mucin-2*) in the jejunum, ileum, and colon (Fig. 1H and I). d-lactate and diamine oxidase (DAO) reflect intestinal barrier integrity. The results showed that the B. animalis treatment also did not affect the contents of serum d-lactate and DAO (Fig. 1J and K).

### Effects of Bifidobacterium animalis on jejunum mucosal enzyme activities.

In the jejunum, supplementation of B. animalis to the diet did not influence the activities of trypsin, pepsin, and lipase (Table 7). However, dietary supplementation with B. animalis increased the amylase activity (*P < *0.05).

**TABLE 7:** Effects of Bifidobacterium animalis on jejunum mucosal enzyme activities of weaning pigs

Items	Con^*a*^	Bif^*a*^	SEM^*a*^	*P* value
Trypsin, U/mg protein	86.63	79.5	5.20	0.526
Pepsin, U/mg protein	1.55	1.49	0.10	0.780
Lipase, U/mg protein	9.99	9.27	0.46	0.473
Amylase, U/mg protein	19.65	27.18	1.98	0.049

a Con, Control group; Bif, Bifidobacterium animalis group; SEM, standard error of the means. *n* = 5 for each group.

### Effects of Bifidobacterium animalis on intestinal inflammation.

**FIG 2:**
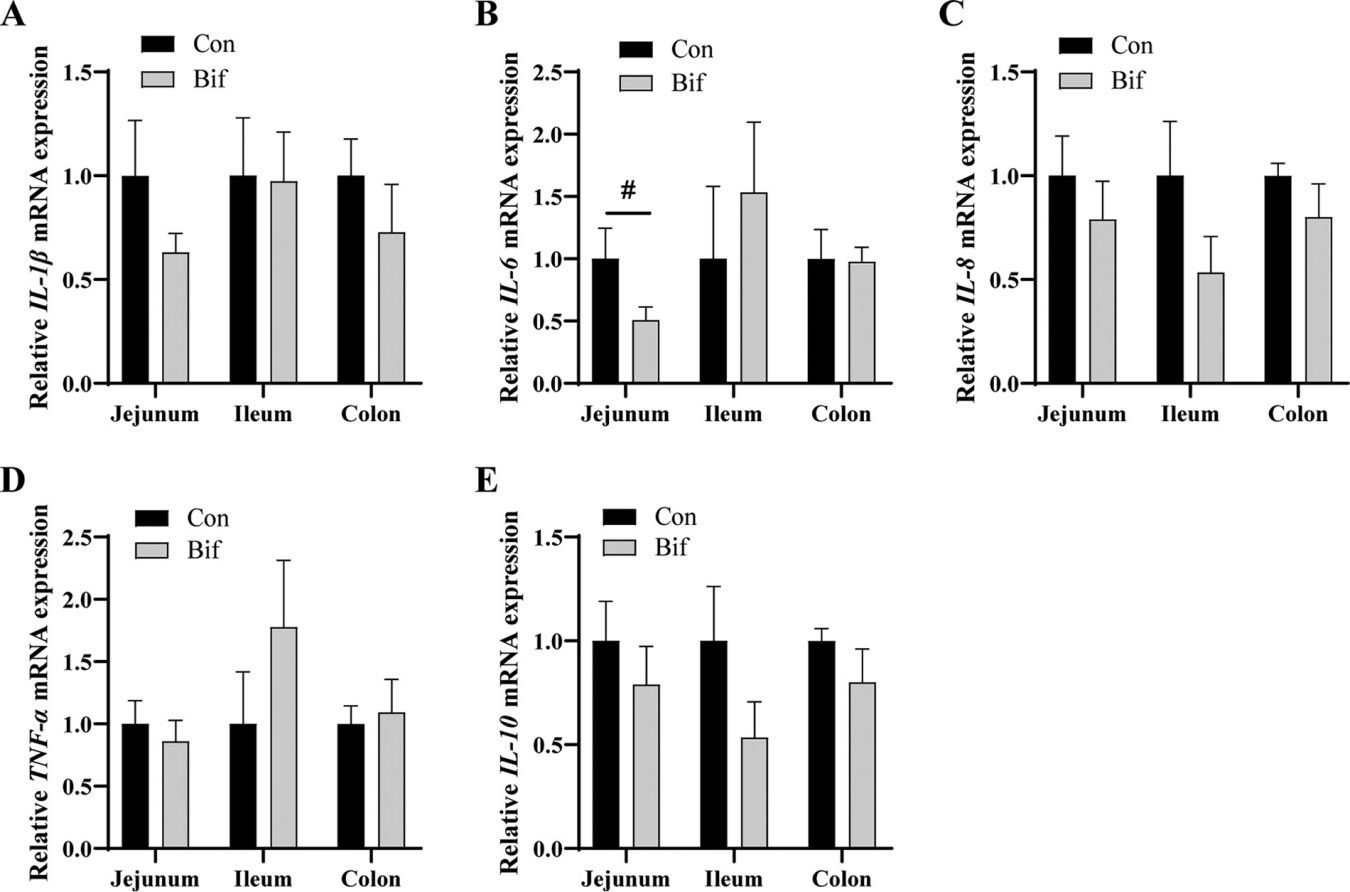
The effects of Bifidobacterium animalis on intestinal inflammatory genes expression. The relative mRNA expression of *IL-1β* (A), *IL-6* (B), *IL-8* (C), *TNF-α* (D), and *IL-10* (E). Con, Control group; Bif, Bifidobacterium animalis group; #, significant trend. SEM, standard error of the means. *n* = 5 for each group.

The inflammatory genes’ relative mRNA expression of different segments in the intestine are shown in Fig. 2. There were no remarkable effects on the relative mRNA expression of *IL-1β*, *IL-6*, *IL-8*, tumor necrosis factor α (*TNF-α*), and *IL-10* in the intestine between the two groups (Fig. 2A to E). Compared with the Con group, B. animalis tended to decrease the relative mRNA expression of *IL-6* in the jejunum (Fig. 2B, *P = *0.10).

### Effects of Bifidobacterium animalis on intestinal microbiota of weaning piglets.

**FIG 3:**
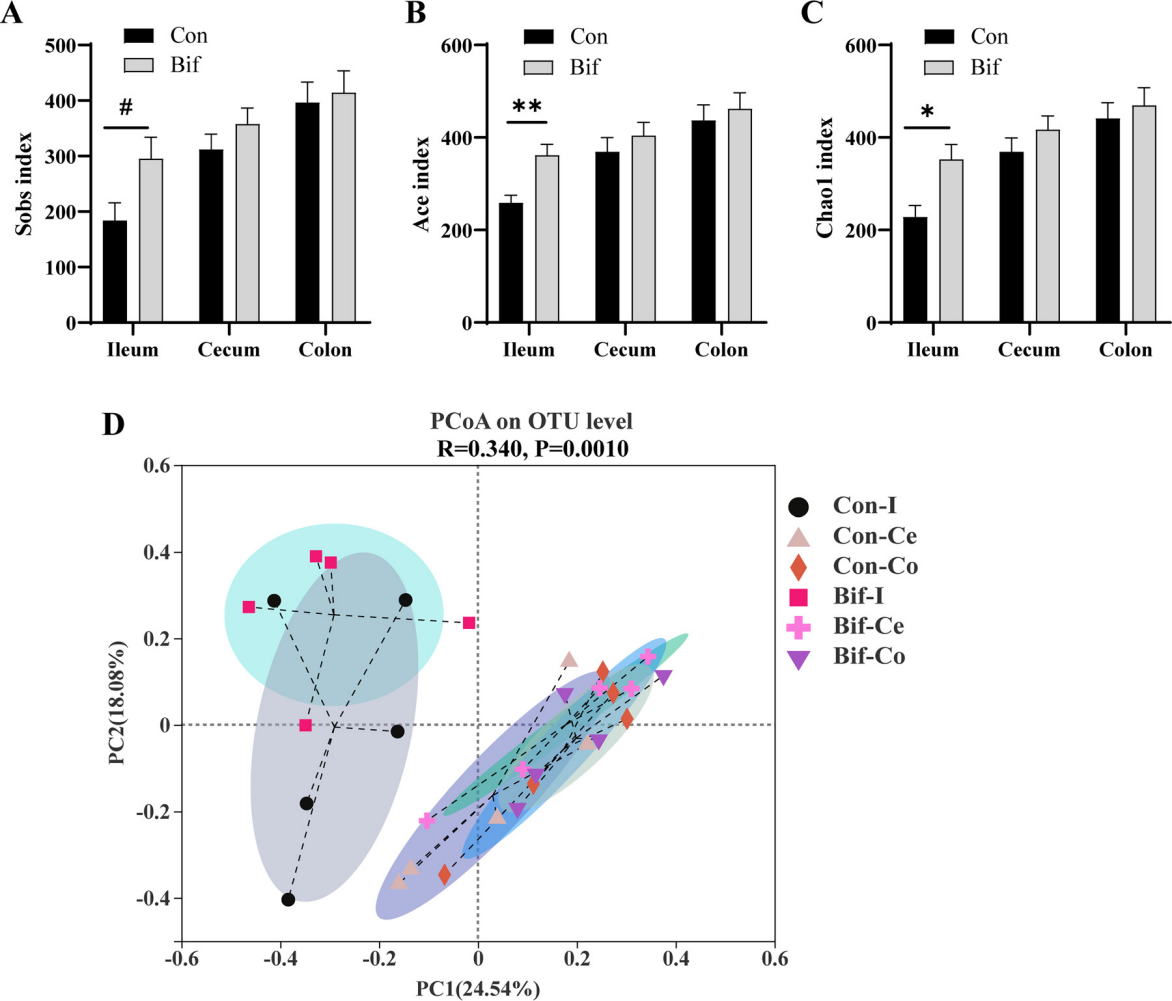
The intestinal digesta microbiota diversities between the Bif and Con piglets. (A–C) Alpha diversity of Sobs (A), Ace (B), and (C) Chao1 indices; (D) principal coordinate analysis (PCoA) of ileal, cecal, and colonic digesta samples in the Bif and Con groups based on Bray-Curtis distances. Data are means ± SEM. *, *P < *0.05; **, *P < *0.01; #, significant trend *P < *0.10. *n* = 5 for each group. Con, Control group; Bif, Bifidobacterium animalis group; Con-I, ileal digesta in control group; Con-Ce, cecal digesta in control group; Con-Co, colonic digesta in control group; Bif-I, ileal digesta in Bifidobacterium animalis group; Bif-Ce, cecal digesta in Bifidobacterium animalis group; Bif-Co, colonic digesta in Bifidobacterium animalis group.

Next, the differences in microbiota composition in various segments of intestinal digesta samples were analyzed (Fig. S1A and B in Supplemental File 1). Alpha diversity (describing the diversity of populations within a sample) indices were calculated to assess the richness and diversity of bacterial communities. The richness of the bacterial community was evaluated by Ace, Chao1, and Sobs indices (Fig. 3A to C), and the diversity of the bacterial community was estimated by Shannon and Simpson indices (Fig. S2A and B in Supplemental File 1). Pigs in the Bif group had higher Ace and Chao1 indices in ileal digesta than pigs in the control group (*P < *0.05, Fig. 3B and C). The Sobs index of ileum in the B. animalis-treated piglets displayed an increasing trend (*P = *0.06, Fig. 3A). No effects were observed for alpha diversity indices in the hindgut between the two groups (Fig. 3A to C and Fig. S2A and B in Supplemental File 1). Beta diversity (describing the differences between populations among different samples) was assessed by principal component analysis (PCoA) plots based on Bray-Curtis distances. Beta diversity indicated no obvious separation in microbiota composition structure between the Bif and the Con groups in the same segment of the intestine (Fig. 3D, Fig. S2C to E in Supplemental File 1).

**FIG 4:**
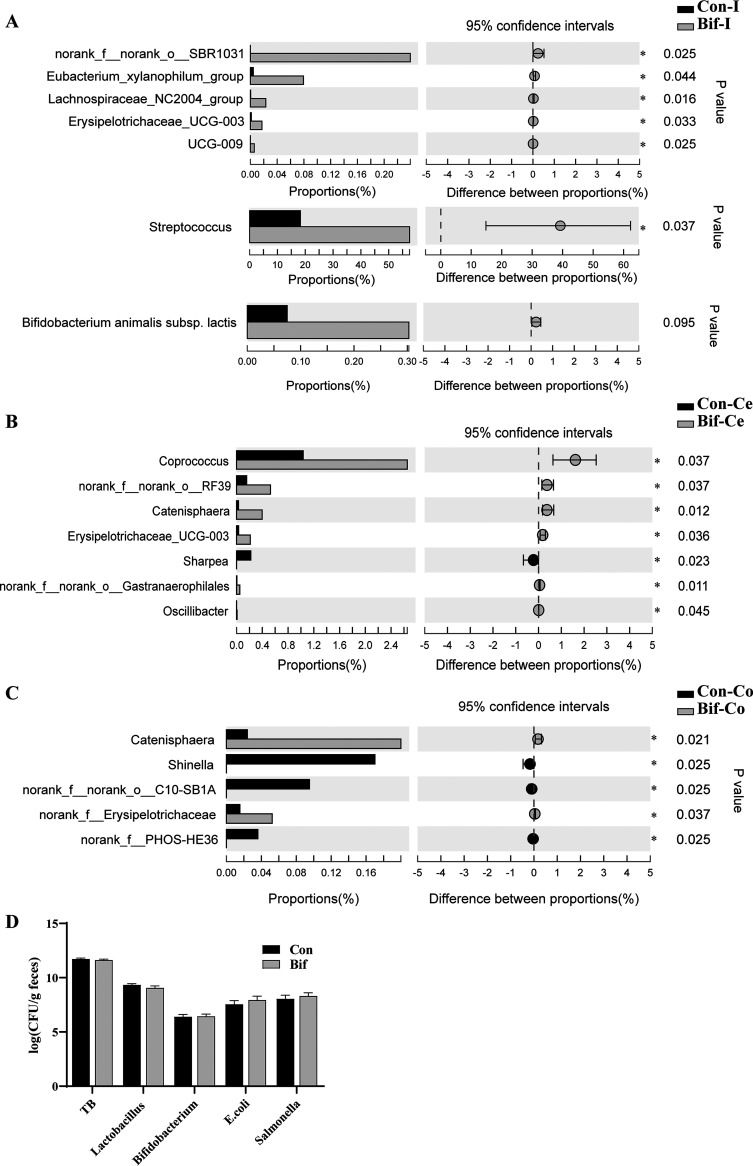
The differential digesta bacteria between the Con and Bif group pigs. The differential bacteria from the ileal (A), cecal (B), and colonic (C) digesta samples between the Con and the Bif pigs (*n* = 5); (D) the absolute copy numbers of specific bacteria in the fecal samples (*n* = 8). Data are means ± SEM. *, *P < *0.05. Con, Control group; Bif, Bifidobacterium animalis group; Con-I, ileal digesta samples in the control group; Con-Ce, cecal digesta samples in the control group; Con-Co, colonic digesta samples in the control group; Bif-I, ileal digesta samples in Bifidobacterium animalis group; Bif-Ce, cecal digesta samples in Bifidobacterium animalis group; Bif-Co, colonic digesta samples in Bifidobacterium animalis group. TB, total bacteria.

The relative abundances of genera Streptococcus and *Erysipelotrichaceae_UCG-003* were increased in the ileal digesta of pigs fed the B. animalis diet compared with those fed the control diet (*P < *0.05, Fig. 4A). In the cecal digesta, the relative abundances of *Coprococcus*, *Erysipelotrichaceae_UCG-003*, and *Oscillibacter* were elevated in pigs from the Bif group (*P < *0.05, Fig. 4B). B. animalis supplementation promoted the relative abundances of *Catenisphaera* in the cecal and colonic digesta (*P < *0.05, Fig. 4B and C). Importantly, the abundance of Bifidobacterium animalis subsp. lactis from ileal digesta in the Bif group showed an increasing trend compared to that in the Con group (*P = *0.095, Fig. 4A). Next, real-time quantitative PCR was used to measure the absolute copy numbers of specific bacteria from the feces. The results revealed that there were no markable changes for the copies of total bacteria (TB), *Lactobacillus*, *Bifidobacterium*, Escherichia coli, and Salmonella from the feces between the two groups (Fig. 4D).

### Effects of Bifidobacterium animalis on mucosal microbiota of weaning piglets.

**FIG 5:**
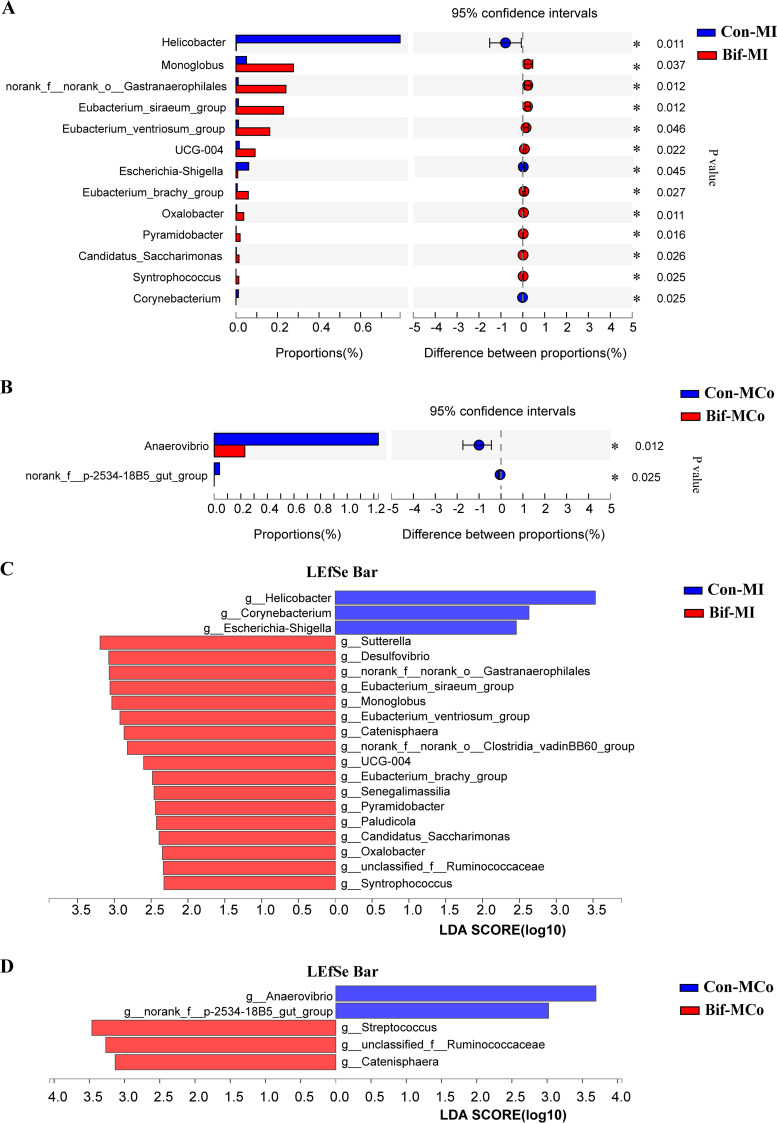
The differential mucosa bacteria between the Con and Bif group pigs. The differential bacteria from the ileal (A) and colonic (B) mucosa samples between the Con and the Bif pigs. Histograms of Linear discriminant analysis (LDA) score at the genus level in ileal (C), and colonic (D) mucosa samples between the Con and the Bif pigs. LDA score >2, *n* = 5 for each group. Con-MI, ileal mucosa samples in control group; Bif-MI, ileal mucosa samples in Bifidobacterium animalis group; Con-MCo, colonic mucosa samples in control group; Bif-MCo, colonic mucosa samples in Bifidobacterium animalis group. *, *P < *0.05.

The mucosal microbiota was closely related to animal health. The mucosal microbiota in the ileum and colon were also analyzed (Fig. S1C and D in Supplemental File 1). The results showed that neither alpha diversity indices nor beta diversity had differences from ileal and colonic mucosa-associated microbiota between the Con and the Bif groups (Fig. S3 in Supplemental File 1). Differential bacteria analysis at the genus level found that the relative abundances of *Helicobacter* and Escherichia-*Shigella* were reduced in the ileal mucosa of pigs from the Bif group compared with those from the Con group (Fig. 5A, *P < *0.05). Linear discriminant analysis (LDA) effect size (LEfSe) analysis was used to identify intergroup differential bacteria. Consistently, LEfSe analysis exhibited that the dietary addition of B. animalis also declined the *Helicobacter* and Escherichia-*Shigella* abundances of ileal mucosa-associated microbiota (Fig. 5C, LDA > 2). Additionally, *Anaerovibrio* was enriched in colonic mucosa samples of pigs from the Con group (Fig. 5B and D, *P < *0.05)., whereas Streptococcus and *Catenisphaera* populations were enriched in colonic mucosa samples of pigs from the Bif group (Fig. 5D, LDA > 2).

## DISCUSSION

Probiotics have been extensively studied in animal production. Lactic acid bacteria, especially the *Lactobacillus* and *Bifidobacterium* species, exhibit various beneficial effects on hosts. Previous studies indicated that supplementation of *Lactobacillus* or *Bifidobacterium* improved the growth performance of weaning piglets and decreased diarrhea incidence by regulating the immune function, enhancing the epithelial barrier, increasing the antioxidant capability, and inhibiting the growth of pathogens (10, 18–20). In this study, we investigated the effects of B. animalis on the growth performance of weaning piglets. The results showed that dietary supplementation of B. animalis increased the final BW and ADG on day 15 to 28 and the whole experimental period of weaning pigs, but the FCR significantly decreased from day 15 to 28 and the whole period. Additionally, the pigs fed a diet with the addition of B. animalis reduced the incidence of diarrhea compared with the control group. The effects of Bifidobacterium animalis strains on growth performance in weaning pigs have a few reports. Modesto et al. (21) found that the ADG had a linear increase with increasing dietary levels of B. animalis strain Ra18 in weaning pigs for the Salmonella Typhimurium infection model. The diet supplementation of the probiotic mixture, containing Enterococcus faecium, B. animalis, and Lactobacillus salivarius, increased BW and body weight gain (BWG) on days 21, 28, 35, and 42 but markedly decreased the FCR at related time points in chicken for the *Eimeria* infection model (22). Broilers receiving *in ovo* inoculation with B. animalis improved BW and BWG and decreased the FCR on days 1 to 28, while no difference in ADFI was observed between the B. animalis and control groups (23). Similarly, a recent study found that rabbits received 1 × 10 ^9^ CFU Bifidobacterium animalis subsp. lactis BB-12 (B. animalis BB12) for 30 days had remarkably increased final BW on day 60 in comparison with the control group (24). Meanwhile, B. animalis BB12 also declined the FCR in rabbits (24). Importantly, the amylase of jejunum in the Bif group was higher than in the control group. Numerous studies demonstrated that probiotics can improve the digestibility of dry matter, energy, crude protein, and crude fiber in weaning piglets (8, 10). Jin et al. (25) indicated that a lactobacillus mixture promotes amylase activities in broiler chickens. Thus, the improvement of dietary nutrient digestibility in pigs fed with probiotics may be explained by increased enzyme production and activity (8).

A mechanism of its health-promoting effects on animals is that probiotics could enhance immune function and suppress inflammatory reactions (10). Our results showed that B. animalis failed to affect the levels of immunoglobulin and inflammatory cytokines in serum or the relative inflammatory genes expression in the intestine, whereas the relative *IL-6* mRNA expression in jejunum had a decreased tendency in B. animalis*-*treated pigs. Similarly, the serum contents of IgA, TNF-β, and IL-1β were not changed in B. animalis-treated broilers compared with the control group (26). In addition, our results were consistent with a recent report, which showed that there were no differences in serum levels of IL-8, TNF-α, and IL-10 or in different segments of the intestine (jejunum, ileum, and colon) between the germfree piglets and B. animalis BB12-treated germfree piglets (13).

The antioxidant enzymes, such as CAT, T-AOC, T-SOD, and GSH-Px play a pivotal role in eliminating oxidative damage and maintaining cell structure. The MDA is regarded as an important indicator of lipid peroxidation. Thus, these parameters are often used to reflect animal health and oxidative stress status. Under stress conditions, the activity of CAT, T-AOC, T-SOD, and GSH-Px was reduced, whereas the level of MDA increased (27). The growing evidence indicated that probiotics, especially lactic acid bacteria, possessed an antioxidant effect (19). Pigs fed a diet supplementation of Lactobacillus plantarum (L. plantarum) had increased contents of SOD and GSH-Px in serum, while the concentration of MDA was decreased (28). In the present study, no differences were observed in serum activities of antioxidant enzyme activities and level of MDA between the control and B. animalis pigs. However, B. animalis treatment significantly elevated the T-AOC activity and reduced the MDA level in the jejunum mucosa. In consistency with our findings, previous studies reported that B. animalis secretion of selenium-contained protein exhibited strong antioxidant activity *in vivo* (16, 29). Meanwhile, B. animalis culture supernatant or cells could significantly enhance the activities of antioxidant enzymes and reduce MDA levels in the d-galactose-induced-aging mice model (29). These results indicated that the beneficial effects of B. animalis on growth performance in weaning piglets might be associated with enhancing antioxidant capacity in the intestine.

Intestinal morphology indicators, such as VH, CD, and VH:CD ratio, are used to evaluate the intestinal mucosal function and are closely related to animal performance. In this study, our results found that B. animalis increased the VH and VH:CD ratio in the duodenum but did not differ in the jejunum and ileum. Barba-Vidal (12) also found that the VH and VH:CD ratio was not affected in B. animalis BPL6 combination with B. longum-treated weaning pigs without Salmonella infection, while B. animalis BPL6 significantly increased the VH:CD ratio on day 8 post Salmonella challenge (12). There was similar result indicated that B. animalis BB12 alleviated ileal epithelial damage post-Salmonella challenge (13).

The beneficial effect of probiotics on intestinal barrier function has also been suggested as a reason for their health-promoting effects on animals (9, 30). Intestinal epithelial tight junction proteins contribute to maintaining cell-cell interaction and determine the primarily paracellular permeability (31). To prevent pathogens infection, intestinal epithelia are covered with a thick and complex mucus layer, which is mainly composed of mucins secreted by goblet cells (32). Our results found that B. animalis did not influence the relative mRNA expression of tight junction molecules (*ZO-1*, *claudin-1*, and *occludin*) and mucin proteins. The serum DAO and d-lactate reflect intestinal permeability, and there were no differences between the Bif and Con groups as well. However, B. animalis-treated pigs markedly increased the number of goblet cells in the jejunum. A recent report demonstrated that the relative mRNA expression of *claudin-1* and *occludin* were not affected in the ileum and colon of B. animalis BB12-treated germfree piglets, respectively, compared to those in the control group (13). Consistently, there was no difference in ileal goblet cells between Bifidobacterium boum RP36 or RP37 strain and control groups germfree piglets (13). It suggested that B. animalis may have a limited effect on intestinal barrier function for animals in normal conditions without pathogen infection. L. plantarum strain did not change the tight junction proteins expression without enterotoxigenic E. coli infection in the IPEC-J2 model, but L. plantarum improved the intestinal epithelial barrier under the E. coli infection (33). B. animalis BB12 treatment increased *claudin-1* protein mRNA expression in the ileum of germfree piglets post Salmonella infection.

Gut microbiota is critical for improving growth performance and defending against pathogens' colonization (2, 34). Our results showed that the lumen of the ileal Ace index and Chao1 index remarkably increased in the Bif group pigs, indicating that dietary supplementation with B. animalis increased the alpha diversity in ileal microbiota. However, B. animalis did not change the bacterial alpha diversity in the hindgut. Notably, there were no differences in beta diversity in the ileum, cecum, and colon between the Bif and Con groups of pigs. Similarly, the alpha diversity indices and structure in ileal and colonic mucosa-associated microbiota were not affected in B. animalis-treated pigs compared with pigs in the control group. At the genus level, supplementation of B. animalis in diet promoted the relative abundances of the ileal lumen of Streptococcus and the cecal lumen of *Coprococcus* and *Oscillibacter*. Additionally, B. animalis supplementation increased the relative abundance of *Catenisphaera* in both lumens of the cecum and colon. Some Streptococcus spp belonging to lactic acid bacteria predominate in pigs as beneficial bacteria (20, 35). In the present study, unclassified Streptococcus may have a beneficial effect on weaning pigs. Similarly, the dietary addition of zinc oxide increased Streptococcus equinus and Streptococcus lutetiensis relative abundances (36). The *Coprococcus* and *Oscillibacter* were considered potentially beneficial bacteria or next-generation probiotics (37–40). Several studies found that *Oscillibacter* was associated with human health, especially inflammatory disease (41). Recent research reported that *Oscillibacter* showed a positive association in the high feed efficiency group of broiler chicken (42). In the current study, dietary supplements with B. animalis promoted *Erysipelotrichaceae_UCG-003* populations in both lumens of the ileum and cecum, which may positively affect the growth performance of pigs. Polysaccharide utilization loci analysis indicated that *Erysipelotrichaceae* strains may have the ability to utilize plant polysaccharides (43). Concordantly, pigs fed a diet with high corn-based arabinoxylans increased *Erysipelotrichaceae_UCG-002* abundance. Interestingly, the cecal relative abundance of the family *Erysipelotrichaceae* showed a negative correlation with FCR in broiler chickens (44). Besides, *Catenisphaera*, a member of the family *Erysipelotrichaceae* maybe also contribute to improving the growth performance of pigs (45). Importantly, dietary supplementation with B. animalis increased the abundance of B. animalis in the ileal digesta and decreased the abundance of potential pathogens *Helicobacter* and Escherichia-*Shigella* in the ileal mucosa. Collectively, these results indicated that B. animalis modulated the gut composition in different intestinal segments and enriched some beneficial bacteria, which consequently improved growth performance. Meanwhile, B. animalis decreased the potential pathogens populations in ileal mucosa.

In conclusion, this study showed that B. animalis increased growth performance, decreased diarrhea incidence, improved duodenal morphology, and enhanced antioxidant capacity in the jejunum of weaning piglets, but did not exert on the intestinal barrier and immune function significantly. Dietary supplementation with B. animalis increased the abundance of B. animalis in the ileum. However, B. animalis supplementation decreased the abundances of *Helicobacter* and Escherichia-*Shigella* might be a growth-promoting attribute. Thus, B. animalis can be considered a potential beneficial bacterium for pigs.

## MATERIALS AND METHODS

### Animals, diets, and experimental design.

A total of 96 (Duroc × Landrace × Large White) weaning piglets with average initial body weight (BW) of 8.16 ± 0.12 kg were randomly divided into 2 groups based on BW and sex. There were 6 replicate pens per treatment and 8 piglets per pen. The control group (Con) received a control diet without supplementation of probiotics. The Bifidobacterium animalis treated group (Bif) was fed a diet with 10^10^ CFU Bifidobacterium animalis subsp. lactis per kg. Bifidobacterium animalis subsp. lactis JYBR-190 was provided by ZhongKe-JiaYi Biological Engineering Co., Ltd. (Shandong, China). The experimental period included two feeding phases: phase 1, from day 1 to day 14 postweaning; phase 2, from day 15 to day 28 postweaning. The dietary compositions and nutrient concentrations are presented in Table 1. All diets without antibiotics were formulated to meet the nutrient requirements of NRC (2012) (46). Pigs were raised in an environmentally controlled room with slatted plastic flooring and a mechanical ventilation system. The environmental temperature was maintained at 24 to 28°C and relative humidity was controlled at 40% to 60%. Pigs were given *ad libitum* access to feed and water throughout the trial for 28 days.

### Samples collection.

Individual pig BW was weighed on days 0, 14, and 28. Pen feed intake was also recorded on days 14 and 28 of the experiment. The average daily gain (ADG), average daily feed intake (ADFI), and feed conversion ratio (FCR) were calculated accordingly. The feces of pigs were scored daily for diarrhea according to the following criteria: 1, firm and well-form feces; 2, soft and form feces; 3, sloppy feces and mild diarrhea; and 4, pasty and liquid diarrhea. The incidence of diarrhea was calculated for the first 7 d after weaning. The incidence of diarrhea was calculated as follows: diarrhea incidence (%) = (total number of pigs per pen with diarrhea)/(number of pigs per pen × 7 d) × 100%.

On day 28, blood samples were collected from the jugular vein (at least one pig per pen) and serum was separated after centrifugation at 3,000 × *g* for 15 min at 4°C. Fresh fecal samples were collected into 2 mL sterile tubes on day 28 and stored at −80°C for further analysis.

On day 29, five pigs from each group were euthanized. The digesta samples from the ileum, cecum, and colon were immediately collected and placed in liquid nitrogen, and then stored at −80°C for further analysis. The different segments of intestinal samples were collected as described previously (47). Segments from the duodenum, jejunum, and ileum (approximately 3 cm long) were flushed with saline, fixed in 4% paraformaldehyde solution, and then embedded in paraffin for analysis of hematoxylin and eosin (HE) and Periodic acid-Schiff (PAS) staining. The mucosal tissues of the jejunum, ileum, and colon were scraped using the glass slide and stored at −80°C for determination of enzyme activities and related gene expression.

### Biochemical analysis.

The contents of serum d-lactate, immunoglobulin A (IgA), IgG, IgM, interleukin-1β (IL-1β), IL-6, and IL-10 were detected by commercial ELISA kits (Jiancheng Co., Ltd., Nanjing, China) according to manufacturer’s instructions. The serum content of diamine oxidase (DAO) was measured using the biochemical kit following the manufacturer’s protocols (Solarbio Co., Ltd., Beijing China). The contents of total superoxide dismutase (T-SOD), catalase (CAT), total antioxidant capacity (T-AOC), malondialdehyde (MDA), and glutathione peroxidase (GSH-Px) capacity in serum were determined using commercial kits (Jiancheng Co., Ltd., Nanjing, China), respectively.

Jejunum mucosal tissues (approximately 100 mg) were weighed, homogenized in ice-cold 1 mL PBS (phosphate buffer solution), and centrifuged at 3,000 × *g* and 4°C for 10 min. The protein concentration of the jejunal supernatants was measured using the bicinchoninic acid (Thermo Fisher, USA) method according to the manufacturer's protocols. The jejunum mucosal antioxidant indices were determined as described above. The activities of trypsin, pepsin, lipase, and amylase were determined using commercial kits (Jiancheng Co., Ltd., Nanjing, China).

### Morphological examination and measurement of intestinal goblet cell numbers.

The segments of the small intestine were sectioned (5 μm). The sections were stained with the HE method or PAS method. The images were captured under a light microscope (Carl Zeiss, Germany). The villus length (VH) and crypt depth (CD) was measured by random measurements of 10 VH and 10 CD per section. The intestinal goblet cell numbers in the field view were counted by a random selection of a field view in each section. The goblet cells are stained red color by PAS.

### RNA extraction and quantification.

Total RNA was extracted from the tissue using TRIzol Reagent (Invitrogen, USA) according to the manufacturer’s protocol. 1.0 μg of RNA was used to synthesize the first-strand cDNA by using a reverse transcription kit (TaKaRa, Dalian, China). The synthesized cDNA was diluted (1:10, vol/vol) with ultrapure-water, and then stored at −20°C for future analysis. The quantitative real-time PCR was performed on Roche LightCycler 96 system (Roche, Sweden) with specific primers (Table 2). The method of 2^−△△Ct^ was used to calculate the relative gene expression and glyceraldehyde-3-phosphate dehydrogenase (*GADPH*) was normalized as the internal control. All samples were measured in duplicate.

### Microbiota analysis.

Total microbial DNA of the digesta and mucosa from the ileum, cecum, and colon was isolated using the QIAamp Fast DNA Stool Minikit (Qiagen, Germany) following the manufacturer’s protocol. The V3-V4 hypervariable regions of bacteria 16S rRNA genes were amplified with universal primers 338F (ACTCCTACGGGAGGCAGCAG) and 806R (GGACTACHVGGGTWTCTAAT). The purified PCR products were equimolarly combined and paired-end sequenced on the Illumina Miseq platform (Illumina, San Diego, CA, USA).

The raw reads were demultiplexed and quality filtered by QIIME (version 1.9) software with the following criteria (i) the lower-quality sequences with a length of <220 nt or >500 nt, an average quality score of <20, and sequences containing >3 nitrogenous bases were removed; (ii) only overlapping sequences longer than 10 bp and the reads without more than 2 nucleotide mismatches in the primer were assembled. The remaining sequences with 97% similarity were clustered into the same operational taxonomic units (OTUs) using UPARSE (version 7.1) and chimeric sequences were removed. The taxonomy of each OTU representative sequence was analyzed by Ribosomal Database Project (RDP) classifier against the SILVA 16S rRNA gene database using a confidence threshold of 70%. The analysis of the alpha diversity was calculated by the MOTHUR program. For the beta diversity, principal coordinate analysis (PCoA) was performed based on Bray-Curtis distances. The data were analyzed on the Majorbio I-Sanger cloud platform (https://cloud.majorbio.com/).

### Quantification of specific bacteria.

Total bacterial DNA was separated from fecal samples as mentioned above. The specific primers of total bacteria, *Lactobacillus*, *Bifidobacterium*, Escherichia coli (E. coli), and Salmonella were presented in Table 2. Standard curves were obtained from constructing standard plasmids containing the 16S rRNA genes. The copies of target bacteria were calculated by corresponding standard curves as described previously (48).

### Statistical analysis.

Data were performed using SAS 9.4 version (Cary, NC, United States). The data of the two groups were analyzed by unpaired Student's *t* test. The incidence of diarrhea was analyzed by χ^2^ tests. A Nonparametric Wilcoxon rank-sum test was performed to compare the microbiota difference between the Con and Bif piglets at different intestinal segments. *P* values were adjusted with a false discovery rate (FDR). The differential bacteria were identified by linear discriminant analysis (LDA) effect size (LEfSe) analysis. The level of statistical significance was *P < *0.05, whereas 0.05 ≤ *P ≤ *0.1 was considered a trend of significant difference. Data are presented as means ± standard error of the means (SEM).

### Ethics statement.

This work was approved by the Care and Use of Experimental Animal Committee of China Agricultural University (CAU No. AW51211202-1-2).

### Data availability.

All raw FASTQ reads for this study can be found in NCBI Sequence Read Archive (SRA) database PRJNA828137.
