# Supplementary material for: Bifidobacterium animalis Promotes the Growth of Weaning Piglets by Improving Intestinal Development, Enhancing Antioxidant Capacity, and Modulating Gut Microbiota
Source: Appl Environ Microbiol. 2022 Oct 27;88(22):e01296-22. doi: 10.1128/aem.01296-22 (PMC9680619; doi:10.1128/aem.01296-22)
Supplement: Supplemental file 1 — Supplemental material. Download aem.01296-22-s0001.pdf, PDF file, 0.5 MB [file aem.01296-22-s0001.pdf]

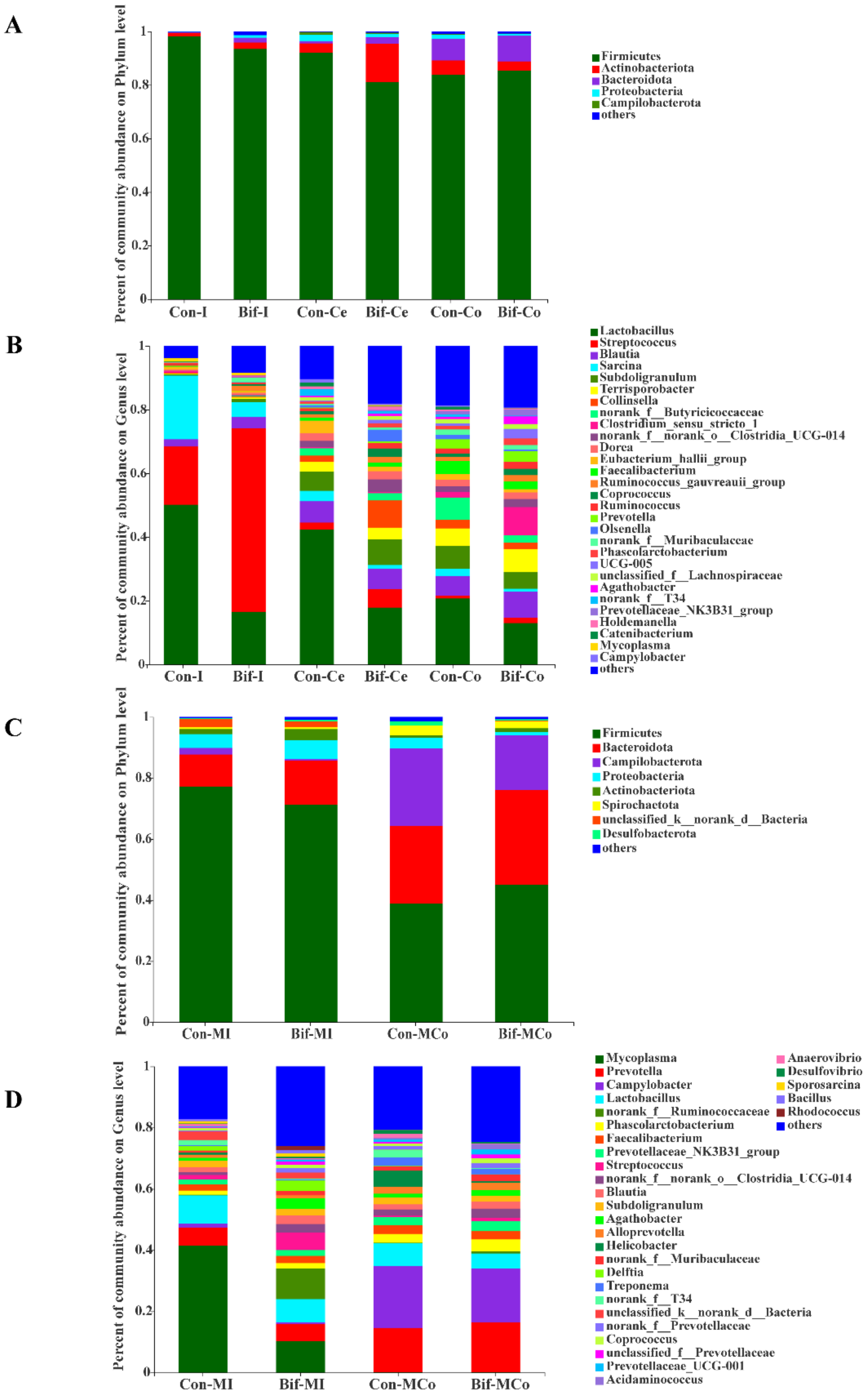

Fig. S1 The intestinal microbiota composition of the Bif and Con piglets. The microbiota composition of digesta samples at the phylum level (A) and the genus level (B) in the Bif and Con groups. The mucosal microbiota composition at the phylum level (C) and the genus level (D) in the Bif and Con groups. Con, Control group; Bif, *Bifidobacterium animalis* group; Con-I, ileal digesta samples in control group; Con-Ce,

cecal digesta samples in control group; Con-Co, colonic digesta samples in control group; Bif-I, ileal digesta samples in *Bifidobacterium animalis* group; Bif-Ce, cecal digesta samples in *Bifidobacterium animalis* group; Bif-Co, colonic digesta samples in *Bifidobacterium animalis* group. Con-MI, ileal mucosa samples in control group; Bif-MI, ileal mucosa samples in *Bifidobacterium animalis* group; Con-MCo, colonic mucosa samples in control group; Bif-MCo, colonic mucosa samples in *Bifidobacterium animalis* group. n=5 for each group.

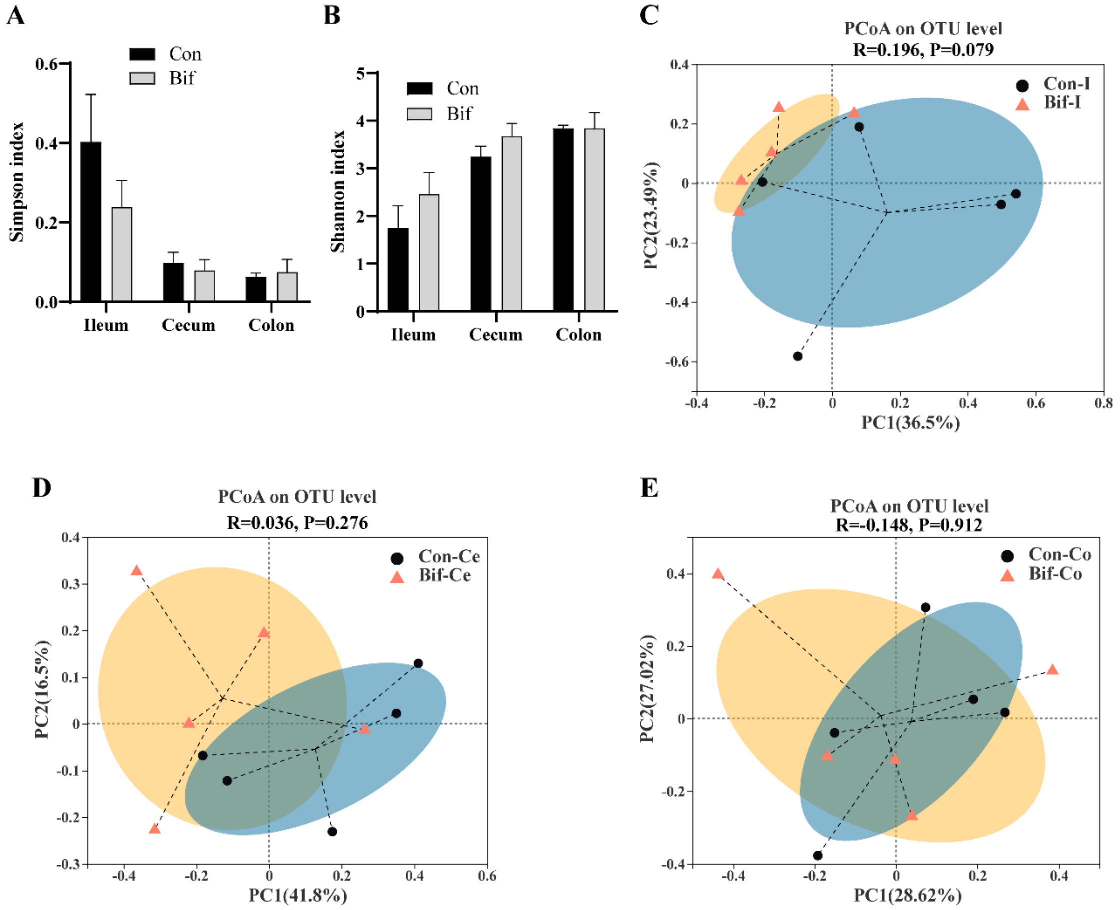

Fig. S2 The diversity of microbiota in lumen of gut. The Simpson (A) and Shannon (B) indices from ileal, cecal, and colonic samples in the two groups; principal coordinate analysis (PCoA) of ileal (C), cecal (D), and colonic (E) digesta samples in the Bif and Con groups based on Bray–Curtis distances. Data are means ± SEM. Groups with no superscript letter or the same superscript letter are not significant ($P > 0.05$); those with different superscript letters are significant ($P < 0.05$). Con, Control group; Bif, *Bifidobacterium animalis* group; Con-I, ileal digesta samples in control group; Con-Ce, cecal digesta samples in control group; Con-Co, colonic digesta samples in control group; Bif-I, ileal digesta samples in *Bifidobacterium animalis* group; Bif-Ce, cecal digesta samples in *Bifidobacterium animalis* group; Bif-Co, colonic digesta samples in *Bifidobacterium animalis* group. n=5 for each group.

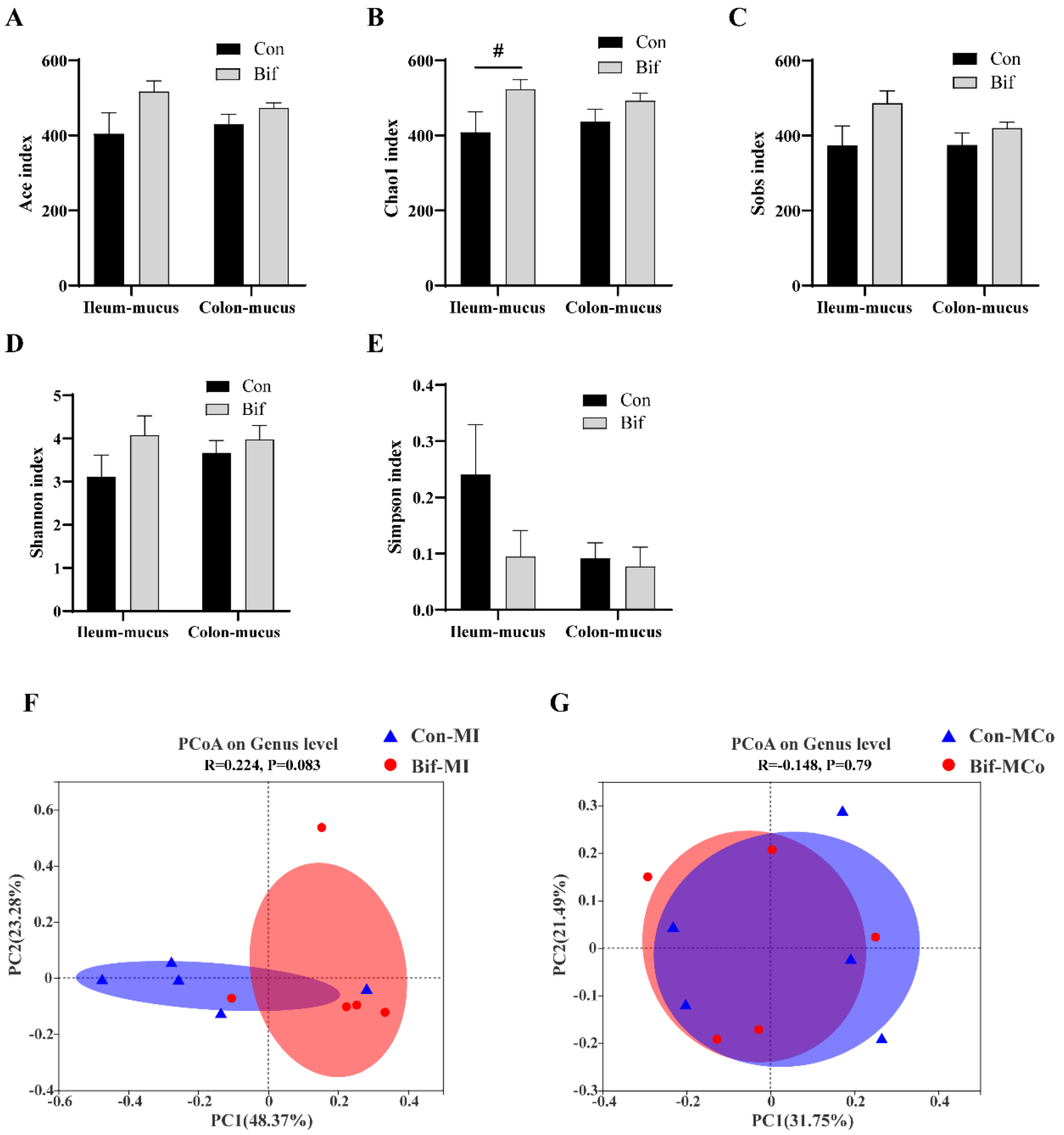

Fig. S3. The diversity of mucosal microbiota. The ACE (A), Chao1 (B), Sobs (C), Shannon (D), and Simpson indices(E) from ileal and colonic mucosa-associated microbiota in the two groups; PCoA of ileal (F) and colonic (G) mucosal-associated microbiota in the Bif and Con groups based on Bray–Curtis distances. # indicates a significant trend, $P < 0.10$. Con-MI, ileal mucosa in control group; Bif-MI, ileal mucosa in *Bifidobacterium animalis* group; Con-MCo, colonic mucosa in control group; Bif-MCo, colonic mucosa in *Bifidobacterium animalis* group. Data are means ± SEM. n=5 for each group.
